# Exploring the Biofilm Formation Capacity in *S. pseudintermedius* and Coagulase-Negative Staphylococci Species

**DOI:** 10.3390/pathogens11060689

**Published:** 2022-06-16

**Authors:** Vanessa Silva, Elisete Correia, José Eduardo Pereira, Camino González-Machado, Rosa Capita, Carlos Alonso-Calleja, Gilberto Igrejas, Patrícia Poeta

**Affiliations:** 1 Microbiology and Antibiotic Resistance Team (MicroART), Department of Veterinary Sciences, University of Trás-os-Montes and Alto Douro (UTAD), 5000-801 Vila Real, Portugal; vanessasilva@utad.pt; 2 Department of Genetics and Biotechnology, University of Trás-os-Montes and Alto Douro (UTAD), 5000-801 Vila Real, Portugal; gigrejas@utad.pt; 3 Functional Genomics and Proteomics Unit, University of Trás-os-Montes and Alto Douro (UTAD), 5000-801 Vila Real, Portugal; 4 LAQV-REQUIMTE, Department of Chemistry, NOVA School of Science and Technology, Universidade Nova de Lisboa, 2829-516 Caparica, Portugal; 5 Center for Computational and Stochastic Mathematics (CEMAT), Department of Mathematics, University of Trás-os-Montes and Alto Douro (UTAD), 5000-801 Vila Real, Portugal; ecorreia@utad.pt; 6 CECAV—Veterinary and Animal Research Centre, University of Trás-os-Montes and Alto Douro (UTAD), 5000-801 Vila Real, Portugal; jeduardo@utad.pt; 7 Associate Laboratory for Animal and Veterinary Science (AL4AnimalS), University of Trás-os-Montes and Alto Douro (UTAD), 5000-801 Vila Real, Portugal; 8 Department of Food Hygiene and Technology, Veterinary Faculty, University of León, E-24071 León, Spain; mgonm@unileon.es (C.G.-M.); rosa.capita@unileon.es (R.C.); carlos.alonso.calleja@unileon.es (C.A.-C.); 9 Institute of Food Science and Technology, University of León, E-24071 León, Spain

**Keywords:** biofilm, staphylococci, coagulase-negative staphylococci (CoNS)

## Abstract

The ability of biofilm formation seems to play an important role in the virulence of staphylococci. However, studies reporting biofilm formation of coagulase-negative staphylococci isolated from animals are still very scarce. Thus, we aimed to evaluate the biofilm-forming capacity of CoNS and *S. pseudintermedius* isolated from several animal species and to investigate the effect of conventional antimicrobials on biofilm reduction. A total of 35 *S. pseudintermedius* and 192 CoNS were included. Biofilm formation was accessed by the microtiter plate assay and the biofilms were stained by crystal violet. Association between biofilm formation and staphylococci species and antimicrobial resistance was also performed. Biofilm susceptibility testing was performed with tetracycline and amikacin at the minimum inhibitory concentration (MIC) and 10 × MIC. The metabolic activity of the biofilm cells after antimicrobial treatment was accessed by the XTT assay. All isolates formed biofilm, with *S. urealyticus* producing the most biofilm biomass and *S. pseudintermedius* producing the least biomass. There was a positive association between biofilm formation and multidrug resistance as well as resistance to individual antimicrobials. Neither tetracycline nor amikacin were able to eradicate the biofilm, not even at the highest concentration used. This study provides new insights into biofilm formation and the effects of antimicrobials on CoNS species.

## 1. Introduction

Staphylococci are divided into two main categories, coagulase-negative staphylococci (CoNS) and coagulase-positive staphylococci (CoPS), based on their ability to induce clotting of mammalian serum [1]. *Staphylococcus lugdunensis* has become known as an “intermediate species” since, although belonging to the CoNS group, it shares several features with CoPS *S. aureus* displaying clinical features of both groups [2]. While *S. aureus* is a classical mammal pathogen and it can cause life-threatening diseases, CoNS were considered to be nearly nonpathogenic [3]. In fact, before the 1970s, CoNS were recognized as contaminants in clinical samples [4]. CoNS are commensal organisms that colonize the skin and mucous membranes of humans and animals; however, in recent years, it has been found that CoNS are also opportunistic pathogens in veterinary and human medicines, which have been established as common causes of many infections [2,3,5]. As stated above, CoNS colonize humans and animals, and although there are more than 50 known species of CoNS, some of them colonize both humans and animals [5,6]. Risk factors for commensal CoNS to cause infection in the host include a compromised immune system, breaking of the natural skin barrier, or the presence of indwelling medical devices [5]. CoNS can colonize specific body parts and cause specific infections; for example, *S. haemolyticus* is involved in native valve endocarditis and *S. saprophyticus* is responsible for up to 10% of urinary tract infections in women [7]. In addition, numerous studies have reported an increase in antimicrobial resistance in CoNS, which limits the therapeutic options [8,9,10]. Regardless of the host, CoNS might be generally prone to increased antimicrobial resistance carriage since CoNS isolated from various animal species also carried resistance to several classes of antimicrobials [11,12,13,14]. In addition, CoNS have the ability to form biofilms on indwelling medical devices, making these infections extremely difficult to treat [15]. Although it was previously thought that CoNS lack the enormous repertoire of virulence factors common in *S. aureus* strains, after molecular and genomic investigations, it appears that CoNS also have a considerable number of genes encoding for biofilm production, adhesion factors hemolysins, and exoenzymes [16].

Biofilms are structured aggregates of bacterial cells that form communities surrounded by an extracellular matrix (ECM) [17]. Staphylococci bacterial cells can attach to biotic and abiotic surfaces through the production of cell wall-anchored proteins of which the microbial surface components recognizing adhesive matrix molecules (MSCRAMMs) are the main family [17,18]. In CoNS biofilms, the bacterial intercellular adhesion is promoted by polysaccharide intercellular adhesin (PIA) [19]. PIA production is dependent upon four genes, *ica*ADBC, encoded by the *ica* operon [17]. Biofilm formation impairs the action of the antimicrobials, disinfectants, and the host immune system; therefore, there is an enhanced resistance. Antimicrobial resistance in biofilm is mainly due to the action of the ECM, which prevents the diffusion of drugs and by the physiological changes in bacteria caused by the differences in the environmental conditions of the biofilm [20]. Biofilm cells can be between 10 and 1000 times more resistant than planktonic cells [21]. Furthermore, in biofilm cells, the mechanisms of resistance are altered and bacteria showing susceptibility to a given compound become quiescent, increasing their tolerance to that compound [22]. Therefore, the aim of this study was to investigate the biofilm formation of CoNS strains isolated from animals (pets, livestock and wild animals). This study also aimed to study the effect of antibiotics commonly used in veterinary medicine on the CoNS biofilm mass reduction and to investigate a possible correlation between staphylococci species, antimicrobial resistance, and biofilm production.

## 2. Material and Methods

### 2.1. Study Design and Bacterial Isolates

Part of this work was a retrospective study that included 226 non-*aureus* staphylococci, namely, 192 CoNS and 35 *S. pseudintermedius* isolates. The CoNS species included *S. lentus* (*n* = 68), *S. sciuri* (*n* = 75), *S. urealyticus* (*n* = 22), *S. vitulinus* (*n* = 6), *S. xylosus* (*n* = 6), *S. haemolyticus* (*n* = 5), *S. epidermidis* (*n* = 3), *S. cohnii* (*n* = 2), *S. succinus*, *S. saprophyticus*, *S. hominis*, and *S. chromogenes*. The isolates were recovered from animals’ infections and from healthy animals between 2018 and 2021: canine pyoderma (31 *S. pseudintermedius*), healthy poultry (36 *S. lentus*, 21 *S. urealyticus*, 15 *S. sciuri*, and 3 *S. haemolyticus*), wild owls (20 *S. sciuri*, 11 *S. lentus*, 2 *S. vitulinus*, 2 *S. epidermidis*, 2 *S. haemolyticus*, 2 *S. xylosus*, 1 *S. saprophyticus*, and 1 *S. succinus*), Miranda donkeys (17 *S. sciuri*, 2 *S. lentus*, 1 *S. xylosus*, and 1 *S. vitulinus*), camels (13 *S. lentus*, 12 *S. sciuri,* 3 *S. xylosus*, 1 *S. epidermidis*, 1 *S. chromogenes*, and 1 *S. hominis*), healthy dogs (5 *S. lentus*, 4 *S. pseudintermedius*, 2 *S. sciuri*, 2 *S. cohnii*, and 1 *S. vitulinus*), and wild hares (9 *S. sciuri,* 2 *S. vitulinus*, 1 *S. lentus* and 1 *S. urealyticus*) [11,23,24,25,26]. All isolates have been previously characterized regarding their antimicrobial resistance and will be used to perform statistical analysis to study the correlations between biofilm formation (evaluated in this study) and antimicrobial resistance of the isolates (Supplementary Table S1) [11,23,24,25,26,27,28,29,30]. *S. aureus* ATCC^®^ 25,923 (quality control strain) was used as a positive control due to its great biofilm-forming capacity. The isolates were cryopreserved at −20 °C in skim milk.

### 2.2. Biofilm Formation Assay

The biofilm formation was investigated by the microtiter assay as previously described, with some modifications [31]. Briefly, two colonies of fresh cultures of staphylococci isolates were transferred to tubes containing 3 mL of Tryptic Soy Broth (TSB, Oxoid, Basingstoke, UK) and incubated at 37 °C for 16 ± 1 h with continuous shaking at 120 rpm (ES-80 Shaker-incubator, Grant Instruments, Cambridge, UK). Then, the standardized staphylococci suspension at 10^6^ cfu/mL was prepared and 200 µL added into each well in the 96-well plate. *S. aureus* ATCC^®^ 25,923 was included in all plates as a positive control. Uninoculated TSB was included as the negative control. The plates were incubated at 37 ºC for 24 h under static conditions. All experiments had seven technical replicates and were performed in triplicate.

#### Biofilm Biomass Quantification

Biofilm mass was quantified using the Crystal Violet (CV) Staining method as previously described by Peeters et al. (2008), with some modifications [32]. After incubation, the plate wells were washed twice with 200 µL of distilled water to remove non-attached bacterial cells and plates were then allowed to dry at room temperature for 2 h. Then, the biofilm cells were fixed with 100 µL of methanol (VWR International) and incubated for 15 min at room temperature. Methanol was removed, and the plates were allowed to dry in a laminar flow cabinet for 10 min. Then, the attached biofilm cells were stained using 100 µL of CV at 1% (*v*/*v*) and were added to each well for 10 min at room temperature. The excess dye was removed by rinsing the plates with distilled water and crystal violet-bound cells were solubilized with 33% (*v*/*v*) acetic acid. The absorbance was measured at 570 nm using a microplate reader BioTek ELx808U (BioTek, Winooski, VT, USA). To standardize the results, the biofilm formation of each isolate was normalized according to the results obtained from the positive-control strain *S. aureus* ATCC^®^ 25923.

### 2.3. Confocal Laser Scanning Microscopy (CLSM)

One isolate of each species was selected for confocal laser scanning microscopy (CLSM) analysis for the visualization of biofilm aggregate structures. The isolates were selected according to their capacity of biofilm formation and antimicrobial resistance. In total, 13 strains representative of the bacterial collection were used.

Biofilm formation was carried out by the microtiter assay. After one hour of adhesion at 37 °C, the wells were washed with 150 mM of NaCl in order to eliminate any non-adherent cells and refilled with 250 µL of TSB. The plates were then incubated for 24 h at 37 °C. After incubation, the wells were washed with 150 mM of NaCl.

For staining with fluorescent dye, 1.00 µL of SYTO 9 (stock 20 mM in DMSO, Thermo Fisher Scientific, Madrid, Spain) was mixed with 1000 µL of NaCl 150 mM, and 250 µL of this solution was added to each well. The plate was then incubated in the dark at 37 °C for 20 min to enable fluorescent labelling of the bacteria.

CLSM image acquisition was performed using a Zeiss LSM 800 Airyscan confocal laser scanning microscope with ZEN 2.3 software (Carl Zeiss, Jena, Germany). Channel mode visualization was done using the 63× (0.8 NA) objective with oil immersion. The microscopic parameters used for the SYTO9-stained cells have been previously reported [33]. Stacks of horizontal plane images (512 × 512 pixels corresponding to 126.8 × 126.8 μm), with a z-step of 0.25 μm, were obtained for each well from three different randomly chosen areas. Three independent experiments were performed for each strain on different days. Original Zeiss files (CZI format) were imported into the IMARIS 9.1 software package (Bitplane, Zurich, Switzerland) for modelling in three dimensions for image analysis. Biovolume represented the amount of biofilm (μm^3^) in the observation field of 16,078.2 μm^2^. Surface coverage (%) reflected the efficiency of substratum colonization by the populations of bacteria. Roughness was an indicator of biofilm heterogeneity since it provided a measure of biofilm thickness. Biofilms of uniform thickness were represented with the value of zero. The maximum thickness (μm) of biofilms was determined directly from the confocal stack images. This experiment was performed in triplicate in three different days.

### 2.4. Effect of Antibiotics on 24 h-Old Biofilms

A total of 21 isolates were selected to investigate the efficacy of conventional antibiotics in reducing biofilm mass. Two isolates of each species were selected according to their biofilm-forming capacity: those that produced the most and the least biofilm of each species, except for *S. saprophyticus*, *S. succinus*, *S. hominis*, *S. chromogenes*, and *S. cohnii*, since only one isolate of each was isolated from animals. Two antibiotics used in veterinary medicine were chosen for this assay: tetracycline and amikacin. The minimal inhibitory concentrations (MICs) were determined by a standard broth microdilution method in sterile 96-well microplates according to the EUCAST guidelines and as described by Silva et al. [11]. Biofilm formation was performed as described in Section 2.2. After obtaining the 24 h biofilms, the medium was aspired and replaced with 200 µL of TSB supplemented with amikacin or tetracycline (to a final concentration at MIC, 5 × MIC, and 10 × MIC) and incubated at 37 °C for 24 h under static conditions. Positive controls were included in all pates by adding TBS without antimicrobials. After incubation with antimicrobials, biofilm mass was quantified using the CV staining method, as described in Section 2.2. All experiments had four technical replicates. Analysis was performed on two independent occasions and four technical replicates for each antimicrobial.

#### Effect of Antibiotics on Metabolic Activity

The effect of antimicrobials on the metabolic activity of biofilms was determined by the XTT colorimetric method. After the incubation period with antimicrobial agents, biofilms were washed twice with 200 µL of 0.9% (*w*/*v*) NaCl solution. A cell proliferation assay kit (XTT Kit, AppliChem Panreac, Barcelona, Spain) was used and the reagents were prepared according to the instructions of the manufacturer. Briefly, the reaction solution was prepared by adding 0.1 mL of PMS (*N*-methyl dibenzopyrazine methyl sulfate) to 5 mL of XTT reagent. Then, 50 μL of the reaction solution was added to each well and the plates were incubated for 5 h and the absorbance was measured with a microplate reader (BioTek ELx808U, Winooski, VT, USA) at a wavelength of 490 nm.

### 2.5. Statistical Analysis

Descriptive statistics of the data are presented as the mean (M) and standard deviation (SD) when appropriate. Skewness and kurtosis coefficients were computed for univariate normality analysis purposes. To determine the association between resistance and multi-resistance phenotypes and resistance to a particular antimicrobial with biofilm formation, a one-way analysis of variance (ANOVA) and Tukey’s pos-hoc and independent samples *t*-tests were performed. All statistical analysis was performed using SPSS (IBM SPSS Statistics 26). Statistically significant effects were assumed for *p* < 0.05.

## 3. Results

### 3.1. Biofilm Formation

**Figure 1:**
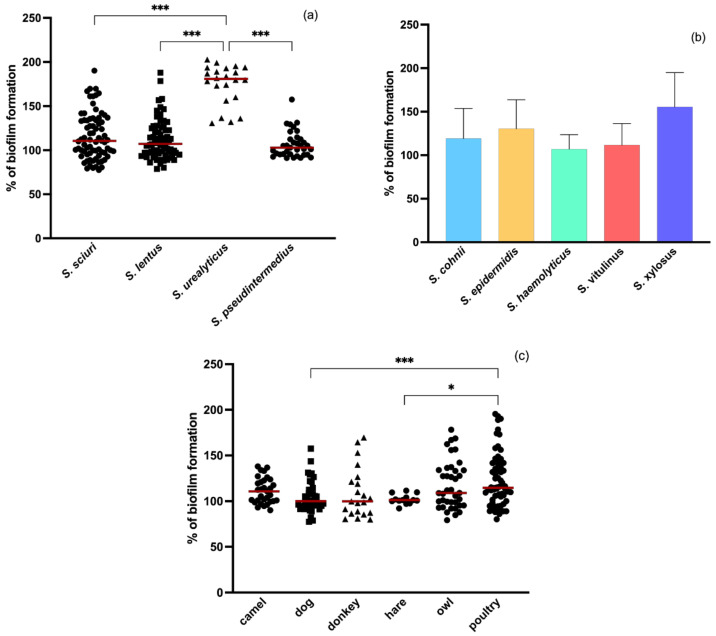
Biofilm-forming capacity of (**a**) the most prevalent staphylococci species; (**b**) the least prevalent species of staphylococci among animal isolates; and (**c**) by animal. The symbols represent the biomass mean of the biofilm formed in independent tests of the individual isolates. The red lines represent the average biofilm mass formed by all isolates. Statistical significance was determined using Tukey’s multiple comparison test (* *p* < 0.05 and *** *p* < 0.001).

A microtiter plate assay was used to measure the biofilm production of 225 non-*aureus* staphylococci, including *S. pseudintermedius* and CoNS, isolated from different animal species. The results were normalized against *S. aureus* ATCC 25,923 (biofilm-producer) so that the comparison of the results could be more consistent. Considered that some staphylococci species had only one or a few associated isolates and in order to have a balanced experimental design in which there is a similar number of isolates in all groups, isolates belonging to the most prevalent species were selected for the statistical analysis. All isolates had the capacity to form biofilm. Figure 1a shows the biofilm formation of each isolate grouped by the most prevalent staphylococcal species. *S. urelyticus* isolates significantly produced more biofilm biomass, with a percentage mean of biofilm formation of 174.7 ± 22.78, than *S. sciuri* (116.1 ± 26.38), *S. lentus* (112.3 ± 22.64) and *S. pseudintermedius* (106.6 ± 14.90) (*p* < 0.001). Among the least prevalent species (Figure 1b), there was no significant differences in biofilm formation (*p* = 0.096), but *S. xylosus* isolates produced the most biofilm. In this study, some CoNS species had only one isolate. The mean percentage of biofilm formation for those isolates was 103.89 ± 3.86, 101.27 ± 5.76, 108.88 ± 7.24 and 138.11 ± 5.09 for *S. chromogenes*, *S. hominis*, *S. saprophyticus* and *S. succinus*, respectively. Among the different animal species included in this study, staphylococci isolated from poultry produced more biofilm biomass than strains isolated from other animals, these differences being significant between isolates from poultry and isolates from hare and dog.

### 3.2. Antimicrobial Resistance and Biofilm Formation

**Figure 2:**
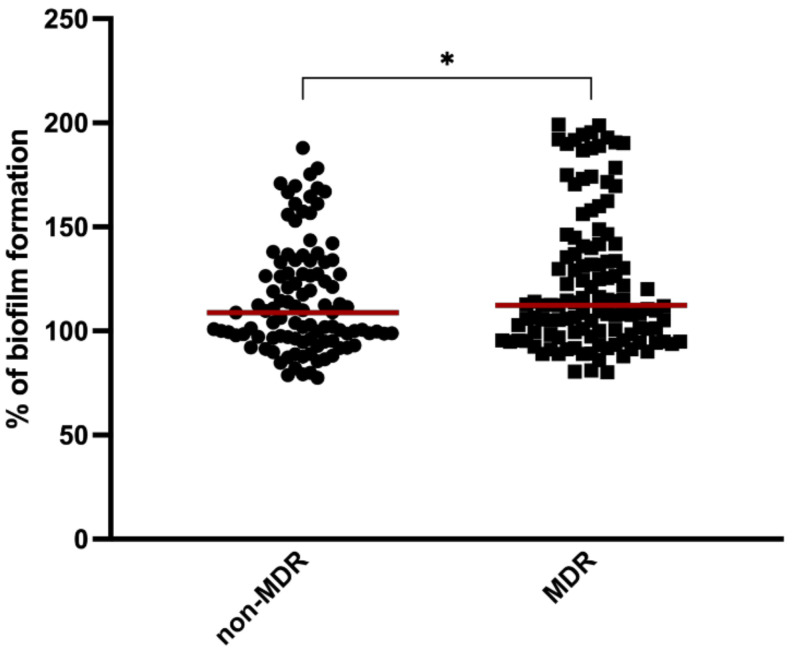
Mean biofilm formation among multidrug-resistant (MDR) and non-multidrug-resistant (non-MDR) isolates. The red lines represent the average biofilm mass formed by all isolates. Statistical significance was determined using Student’s *t*-tests (* *p* < 0.05).

Staphylococci isolates were divided into two categories: multidrug resistant (MDR) and non-multidrug resistant (non-MDR). The relationship between the biofilm-forming capacity and MDR phenotype was investigated by statistical analysis using Student’s *t*-tests. As shown in Figure 2, the MDR strains significantly produced more biofilm than non-MDR isolates (*p* < 0.05). To determine whether biofilm formation is related to resistance to any particular antimicrobial, the biofilm formation was evaluated in isolates with different resistance profiles to 11 antimicrobials (Table 1). The results revealed that isolates resistant to cefoxitin, which is an indicator of methicillin resistance, tetracycline, and fusidic acid produced significantly more biofilm than susceptible isolates (*p* < 0.001). Isolates resistant to erythromycin, clindamycin, and chloramphenicol also formed stronger biofilms than susceptible isolates (*p* < 0.05). In contrast, staphylococci isolates resistant to ciprofloxacin and trimethoprim-sulfamethoxazole produced weaker biofilms when compared to resistant isolates (*p* < 0.05 and *p* < 0.001, respectively).

### 3.3. CLSM Analysis

**Figure 3:**
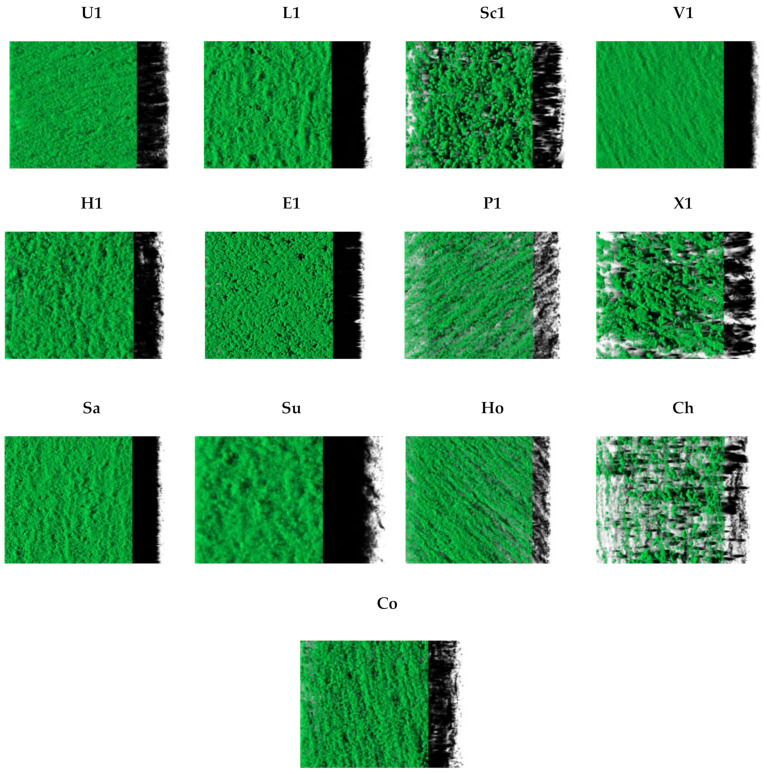
The images correspond to three-dimensional reconstructions obtained by CLSM and processed with IMARIS 9.1 software, including the virtual projections of the shadows on the right. U: *S. urealyticus*; L: *S. lentus*; Sc: *S. sciuri*; V: *S. vitulinus*; H: *S. haemolyticus*; E: *S. epidermidis*; P: *S. pseudintermedius*; X: *S. xylosus*; Sa: *S. saprophyticus*; Su: *S. succinus*; Ho: *S. hominis*; Ch: *S. chromogenes*; Co: *S. cohnii*.

Three-dimensional images of biofilms formed by the 13 different staphylococcal species are shown in Figure 3. In accordance with the results obtained din the microtiter assay, all isolates produced biofilms. Most isolates produced compact biofilm structures that covered the entire available surface, except for *S. sciuri*, *S. pseudintermedius*, *S. xylosus*, and *S. chromogenes*, which produced rough biofilms with irregular coverage and confluent growth areas.

**Figure 4:**
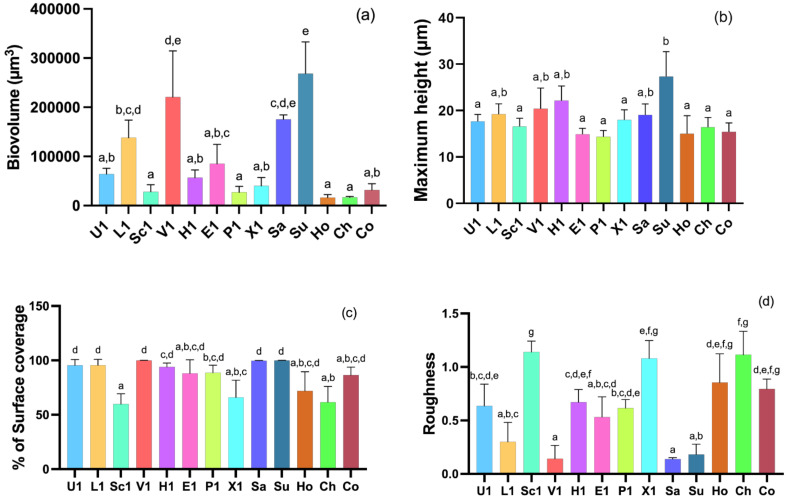
Biovolume in the observation field of 16,078.2 μm^2^ (**a**), maximum height (**b**), maximum height, percentage of surface area covered (**c**), and roughness (**d**) of biofilms formed from the 13 selected CoNS and *S. pseudinteremedius* isolates. Statistical significance was determined using Tukey’s multiple comparison test. The values marked with the same letter are not statistically significant as determined by the Tukey’s post hoc test (*p* < 0.05). U: *S. urealyticus*; L: *S. lentus*; Sc: *S. sciuri*; V: *S. vitulinus*; H: *S. haemolyticus*; E: *S. epidermidis*; P: *S. pseudintermedius*; X: *S. xylosus*; Sa: *S. saprophyticus*; Su: *S. succinus*; Ho: *S. hominis*; Ch: *S. chromogenes*; Co: *S. cohnii*.

The structural parameters, including biovolume, maximum height, percentage of covered surface, and roughness, were obtained from the battery of images acquired by CLSM, which allowed the quantification of biofilm biomass (Figure 4). The results obtained revealed a marked variability in the structure of the biofilms among the CoNS and *S. pseudintermedius* isolates. The biovolume of the biofilms ranges from 16,818.63 ± 2034.19 to 268,342.66 ± 64,584.58 µm^3^ in the observation field of 16,078.2 μm^2^. Accordingly, with the three-dimensional images of biofilms, *S. chromogenes* produced the least biofilm biomass while *S. succinus* produced the most biomass (*p* < 0.001), followed by *S. vitulinus* (220,867.43 ± 93,748.89 µm). Other isolates, such as *S. hominis*, *S. pseudintermedius*, and *S. sciuri*, also produced low amounts of biofilm biomass when compared to other isolates. The greatest biofilm thickness (27.33 ± 5.38 µm) was also observed in *S. succinus* biofilm while the smallest biofilm thickness was detected in *S. pseudintermedius* (14.33 ± 1.38 µm) and *S. epidermidis* (14.92 ± 1.23 µm) (*p* < 0.001). However, regarding the roughness, *S. succinus* biofilm had the lowest scores (0.18 ± 0.10) while *S. sciuri* obtained the highest roughness score (1.14 ± 0.10) (*p* < 0.0001).

### 3.4. Effect of Antimicrobials on 24 h-Old Biofilms

**Figure 5:**
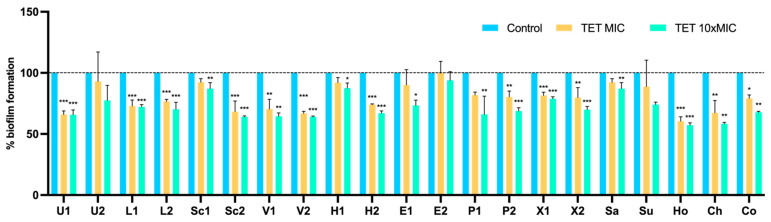
Effect of tetracycline on the biofilm biomass reduction of 21 isolates at concentrations of MIC and 10 × MIC. Data are presented as the mean ± standard deviation for four independent replicates. Statistical significance was determined using Dunnett’s multiple comparison test (* *p* < 0.05; ** *p* < 0.01; *** *p* < 0.001). U: *S. urealyticus*; L: *S. lentus*; Sc: *S. sciuri*; V: *S. vitulinus*; H: *S. haemolyticus*; E: *S. epidermidis*; P: *S. pseudintermedius*; X: *S. xylosus*; Sa: *S. saprophyticus*; Su: *S. succinus*; Ho: *S. hominis*; Ch: *S. chromogenes*; Co: *S. cohnii*; 1: produces the most biofilm; 2: produces the least biofilm.

To assess whether biofilm-specific resistance influences the action of conventional antimicrobials, the MICs of tetracycline and amikacin were determined for 24 h-old biofilms of 21 isolates. The MICs for these isolates ranged from 0.052 to 64 μg/mL for tetracycline and from 0.5 to 64 μg/mL for amikacin. Then, the capacity of these antimicrobials to reduce pre-established 24-h-old biofilms was evaluated using the microtiter biofilm assay at concentrations of MIC and ten times MIC (10 × MIC). Results were normalized according to the 48-h-old biofilm mass recorded for each strain tested grown without the presence of antimicrobials. As shown in Figure 5, biofilms of 18 out of the 21 isolates suffered a significant biomass reduction with 10 isolates having a very highly significant biomass reduction (*p* < 0.001). Although all isolates, except for E2, suffered a biofilm mass reduction when treated with tetracycline at MIC concentration, the reduction was only statistically significant in 13 isolates.

**Figure 6:**
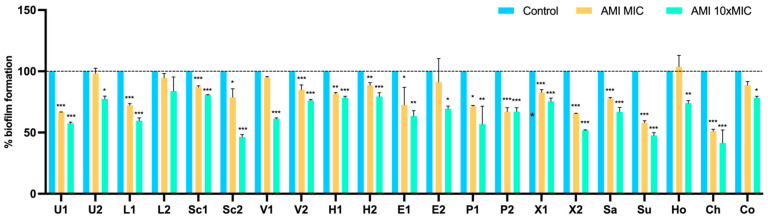
Effect of amikacin on the biofilm biomass reduction of 21 isolates at concentrations of MIC and 10 × MIC. Data are presented as the mean ± standard deviation for four independent replicates. Statistical significance was determined using Dunnett’s multiple comparison test (* *p* < 0.05; ** *p* < 0.01; *** *p* < 0.001). U: *S. urealyticus*; L: *S. lentus*; Sc: *S. sciuri*; V: *S. vitulinus*; H: *S. haemolyticus*; E: *S. epidermidis*; P: *S. pseudintermedius*; X: *S. xylosus*; Sa: *S. saprophyticus*; Su: *S. succinus*; Ho: *S. hominis*; Ch: *S. chromogenes*; Co: *S. cohnii*; 1: produces the most biofilm; 2: produces the least biofilm.

Results for the 24 h-old biofilm treatment with amikacin are shown in Figure 6. After amikacin at 10 × MIC, biofilm mass was reduced in almost all isolates (except for L2), with a highly significant reduction in 15 isolates (*p* < 0.001). At the MIC concentration, amikacin was able to significantly reduce the biofilm of 15 isolates. In contrast, there was an increase in biomass in strain Ho, corresponding to *S. hominis*, after treatment with amikacin at MIC. Nevertheless, this increase was not statistically significant. Overall, tetracycline at 10 × MIC had a higher capacity to reduce biofilm mass than amikacin; however, the difference was not significant (*p* = 0.055).

#### Metabolic Activity

**Figure 7:**
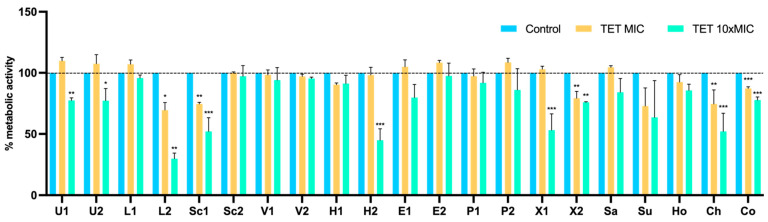
Metabolic activity of staphylococci biofilms before and after treatment with tetracycline at concentrations of MIC and 10 × MIC. The results are expressed as the percentage of metabolic activity. Statistical significance was determined using Dunnett’s multiple comparison test (* *p* < 0.05; ** *p* < 0.01; *** *p* < 0.001). U: *S. urealyticus*; L: *S. lentus*; Sc: *S. sciuri*; V: *S. vitulinus*; H: *S. haemolyticus*; E: *S. epidermidis*; P: *S. pseudintermedius*; X: *S. xylosus*; Sa: *S. saprophyticus*; Su: *S. succinus*; Ho: *S. hominis*; Ch: *S. chromogenes*; Co: *S. cohnii*; 1: produces the most biofilm; 2: produces the least biofilm.

**Figure 8:**
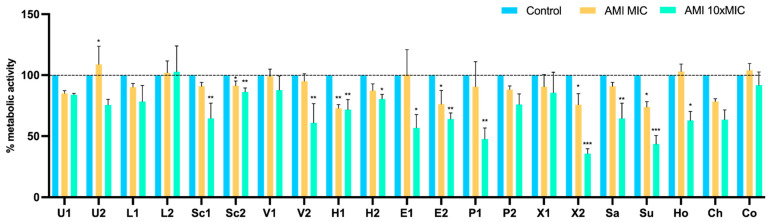
Metabolic activity of staphylococci biofilms before and after treatment with amikacin at concentrations of MIC and 10 × MIC. The results are expressed as the percentage of metabolic activity. Statistical significance was determined using Dunnett’s multiple comparison test (* *p* < 0.05; ** *p* < 0.01; *** *p* < 0.001). U: *S. urealyticus*; L: *S. lentus*; Sc: *S. sciuri*; V: *S. vitulinus*; H: *S. haemolyticus*; E: *S. epidermidis*; P: *S. pseudintermedius*; X: *S. xylosus*; Sa: *S. saprophyticus*; Su: *S. succinus*; Ho: *S. hominis*; Ch: *S. chromogenes*; Co: *S. cohnii*; 1: produces the most biofilm; 2: produces the least biofilm.

The XTT assay was used to measure the cellular metabolic activity as an indicator of cell viability, after treatment with tetracycline and amikacin at concentrations of MIC and 10 × MIC. The results were normalized according to the 48 h-old biofilm of each tested isolate, which were grown without the presence of antimicrobials. After treatment with tetracycline at MIC and 10 × MIC, there was a significant reduction in metabolic activity in 5 and 9 isolates, respectively (Figure 7). In contrast, at the MIC concentration, the biofilm cells of eight isolates increased the metabolic activity. The effect of amikacin on the metabolic activity of biofilms are shown in Figure 8. The metabolic activity of biofilm cells was significantly reduced in 12 isolates at 10 × MIC. In four isolates (U2, L2, Ho, and Co) was also observed an increase in the metabolic activity at the MIC concentration, which corresponds to the results of biomass reduction.

## 4. Discussion

In the last decades, CoNS have emerged as multidrug-resistant nosocomial pathogens constituting a major threat. CoNS species differ greatly from each other and there is no such thing as typical CoNS [34]. Furthermore, although some CoNS species have a specific host, human-associated CoNS species are also regularly isolated from animals and vice versa [5]. Contrary to what was previously thought, CoNS species can cause infections in healthy hosts without apparent risk factors [34]. Furthermore, CoNS infections are often associated with the biofilm formation since these organisms have the ability to colonize abiotic and biotic surfaces [2,35]. Nevertheless, biofilm formation in CoNS species is still not well characterized.

### 4.1. Biofilm Formation

In our study, we investigated the biofilm-forming capacity of *S. pseudintermedius* and 12 CoNS species, including *S. lentus*, *S. sciuri*, *S. urealyticus*, *S. vitulinus*, *S. xylosus*, *S. haemolyticus*, *S. epidermidis*, *S. cohnii*, *S. succinus*, *S. saprophyticus*, *S. hominis*, and *S. chromogenes*. Among all these species, some are primarily human-associated, such as *S. epidermidis*, *S. saprophyticus*, and *S. haemolyticus*, and others are primarily animal associated [5]. All staphylococci had the ability to form biofilm in the plastic surface of the microplate. Among the most prevalent species isolated from animals, *S. urealyticus*, which is an animal-associated species, produced significantly stronger biofilms than the other species. Bino et al. studied the biofilm formation of several CoNS isolated from horses and reported that all species were biofilm producers [36]. In the same study, *S. urealyticus* were classified as strong biofilm producers [36]. Among *S. lentus*, *S. sciuri* and the CoPS *S. pseudintermedius*, there were no significant differences in biofilm production, but all were considered strong biofilm producers since the percentage mean of biofilm production was above 100%. *S. lentus* is often isolated from livestock and people with occupational exposure to livestock while *S. sciuri* has a wider host range [2,37,38,39,40]. In the study of Kala et al., the majority of *S. lentus* and *S. sciuri* isolated from pigs were biofilm producers [41]. *S. pseudintermedius* capacity to form biofilms has been more extensively studied than in the CoNS since they are the main colonizers and pathogens of dogs [23]. Studies have shown that *S. pseudintermedius* have the ability to mostly form medium to strong biofilms [42,43]. Other factors may influence biofilm production such as antimicrobial resistance and clonal lineage of the isolates. After performing multilocus sequence typing, Osland et al. showed that *S. pseudintermedius* belonging to sequence type (ST) 71 former stronger biofilms than strains belonging to other STs. Our *S. pseudintermedius* isolates collected from canine infections belonged to ST123, which differs from ST71 by a one-point mutation on the sar locus, which may explain the high capacity of our isolates to form biofilm. Among the least prominent CoNS species included in this study, *S. heamolyticus* produced less biofilm biomass and *S. xylosus* produced more biomass than the other CoNS. However, these differences were not statistically significant. Previous studies regarding the biofilm formation of *S. haemolyticus* have yielded different results among them, with studies reporting high rates of biofilm formation and others low to medium rates [44,45]. A study conducted with milk samples showed that *S. xylosus* was moderate to strong biofilm formers whereas *S. epidermidis* had the lowest ability to form biofilms [46]. Another study investigated the biofilm-forming ability of CoNS isolated from horses and the highest values were measured for *S. xylosus* strains [36].

### 4.2. Antimicrobial Resistance and Biofilm Formation

There are studies showing an association between antimicrobial resistance and the biofilm-forming capacity of staphylococci strains [47,48]. However, there are also other studies that did not find any association [9]. In our study, MDR staphylococci produced more biofilm biomass than non-MDR isolates (*p* < 0.05). Therefore, we also investigated if the antimicrobial resistance to individual antibiotics influenced the biofilm production. Strains resistant to cefoxitin, and therefore, resistant to methicillin, and to tetracycline produced more biofilm biomass than susceptible isolates (*p* < 0.001). These results may show the importance of individual antimicrobial resistance in the pathogenesis of biofilm-producing strains. Sheikh et al. also found an association between biofilm formation and antimicrobial resistance to most antimicrobials classes, except for oxazolidinones [49]. Koksal et al. showed that methicillin resistance was significantly higher in biofilm-positive isolates [50]. Studies investigating the association between biofilm formation and resistance antimicrobials in CoNS isolates are very scarce. Nevertheless, studies conducted with *S. aureus* also reported a positive relationship between biofilm formation and tetracycline and cefoxitin resistance [51,52,53].

### 4.3. Effect of Antimicrobials on 24 h-Old Biofilms

The biofilm matrix can prevent antimicrobials from entering and reaching their molecular targets by different mechanisms, such as modification/degradation of the antimicrobial, drug tolerance, chelation, and precipitation [54]. It is very important to investigate the biofilm resistance to antimicrobials since the values of MIC do not generally correlate with the minimal biofilm eradication concentration (MBEC). The biofilm formation mechanisms and the effect of antimicrobials on staphylococci have been mainly investigated in *S. lugdunensis* and *S. epidermidis* but little is known about other CoNS species [34]. In our study, we evaluated the biofilm reduction in 12 species of CoNS using tetracycline and amikacin at concentrations of MIC and 10 × MIC. As excepted, none of the antimicrobials, even at high concentrations, were able to eliminate staphylococci biofilms. Tetracycline at 10 × MIC was able to significantly reduce the biofilm mass of 18 isolates except for one *S. urealyticus*, one *S. epidermidis*, and *S. succinus* isolates. Not much is known about the biofilm resistance of *S. urealyticus* and *S. succinus* biofilms but studies on *S. epidermidis* have shown that concentrations higher than 10 × MIC are necessary for biofilm eradication [55,56]. In a recent study, the biofilm biomass of *S. epidermidis* suffered a 55% reduction after treatment with 10 × MIC [55]. Moreover, in the same study, 24 h-old biofilms of *S. chromogenes* and *S. haemolyticus* were also reduced by 30% and 29%, respectively, which are in accordance with our results [55]. Brady et al. have also showed that MBEC concentrations were 10–1000 times greater than that of the MIC breakpoints, with MBEC for tetracycline surpassing 256 µg/mL [56]. Accordingly, in the study by Flemming et al., the isolates tested under planktonic growth conditions were susceptible to tetracycline, but even after the biofilm treatment with 500 mg/L of tetracycline, a significant number of living cells were still detected [57]. Regarding the efficacy of amikacin, almost all isolates’ biofilm suffered a significant reduction in biomass after treatment with 10 × MIC. However, at the MIC concentration, there was an enhancement of biofilm production of *S. hominis*. The biofilm reduction exceeded 50% in *S. sciuiri*, *S. succinus*, and *S. chromogenes*. A study have reported amikacin MBECs between threefold to 1000-fold higher than the MIC in staphylococci biofilms [58,59]. Amikacin belongs to the aminoglycosides class of antimicrobials and some studies reporting the effect of other aminoglycosides on biofilm reduction have shown that even high doses of these antimicrobials, even higher than 1024 µg/mL, were not sufficient to eliminate CoNS biofilms [60,61]. In a study conducted with *S. pseudintermedius*, a >667-fold difference between the MIC and MBIC was observed [62]. The antimicrobial molecule and the mechanism of action can also play a role in the action of antimicrobials upon biofilms. Therefore, the penetration of amikacin in biofilms may be difficult since aminoglycosides are large polar molecules [63]. Our results also show the failure of amikacin and tetracycline to eradicate biofilms is completely independent of any staphylococci species or origin. These differences found between MIC and MBEC may explain the frequent failure in the treatment of CoNS infections with conventional antibiotics [60]. In fact, it is known that bacteria within biofilms are shielded against the action of antimicrobials due to the matrix that serves as a barrier hampering antimicrobial penetration, the reduced metabolic functions of the biofilm cells, the elevated mutation rates of staphylococci within biofilms, and antimicrobial tolerance [34,64,65].

The CV method to quantify biofilm biomass has been extensively used and is based on the CV bond negatively charged surface molecules and polysaccharides in the extracellular matrix of biofilms. However, in this method both dead and alive cells are stained [32]. Therefore, after antimicrobial treatment, we measured the metabolic activity of viable cells using the XTT method. Most biofilm isolates that suffered a significant biomass reduction also showed a lower metabolic activity. A study conducted by Flemming et al. have reported that biofilm treated with tetracycline had a lower metabolic activity even though a significant number of biofilm cells were still alive [57]. However, the metabolic activity of some strains, particularly those that did not undergo a significant reduction, was enhanced at the MIC concentration. This may be due to an increase in the number of viable cells, an increase in the metabolic activity of cells—in an attempt to resist the antimicrobial action—or the cells might have been at the proliferative stage, with a reduced extracellular matrix [64].

## 5. Conclusions

Our study showed that all CoNS and *S. pseudintermedius* isolated from animals are able to form biofilms. *S. urealyticus* strains, which had been isolated from poultry for human consumption, produced more biofilm than other staphylococcus species. We also found an association between biofilm-forming capacity and antimicrobial resistance, particularly to important antimicrobials such as cefoxitin. Our study also reinforces previous findings that CoNS in a biofilm mode are highly resistant to antimicrobials. Neither amikacin nor tetracycline at 10 × MIC were able to eliminate biofilms. Therefore, research for new antimicrobial classes or alternatives to antimicrobials is urgently needed. These staphylococci strains with zoonotic potential had a high capacity to form biofilms and may pose a threat to human health. Thus, this underlines the need for implementation of new measures in the public health and veterinary sectors to prevent transmission of CoNS in the One Health context.

## Figures and Tables

**Table 1:** Mean (M), standard deviation (SD), and univariate effects on biofilm formation when susceptible and resistant to each antibiotic.

Antibiotic	Resistant M ± SD	Susceptible M ± SD	*p*
Penicillin	123.469 ± 38.554	116.446± 28.214	0.262
Cefoxitin	134.263 ± 47.678	115.547 ± 26.949	<0.001
Ciprofloxacin	112.162 ± 22.167	123.607 ± 37.029	0.016
Gentamycin	121.581 ± 43.431	122.230 ± 35.592	0.462
Tobramycin	121.041 ± 37.159	122.504 ± 36.848	0.398
Kanamycin	116.791 ± 38.061	123.691 ± 36.453	0.120
Erythromycin	127.907 ± 43.901	116.695 ± 27.816	0.11
Clindamycin	127.057 ± 42.489	116.182 ± 27.673	0.014
Tetracycline	131.639 ± 44.944	114.379 ± 26.382	<0.001
Chloramphenicol	129.674 ± 42.052	116.704 ± 27.575	0.039
Fusidic acid	140.587 ± 48.318	113.390 ± 25.542	<0.001
Trimethoprim- sulfamethoxazole	103.112 ± 13.315	125.025 ± 37.153	<0.001

## Data Availability

Not applicable.

## Supplementary Materials

The following supporting information can be downloaded at: https://www.mdpi.com/article/10.3390/pathogens11060689/s1, Table S1: Characteristics of the S. pseudintermedius and CoNS isolated from pets, livestock and wild animals.
