# Supplementary material for: Exploring the Biofilm Formation Capacity in S. pseudintermedius and Coagulase-Negative Staphylococci Species
Source: Pathogens. 2022 Jun 16;11(6):689. doi: 10.3390/pathogens11060689 (PMC9229561; doi:10.3390/pathogens11060689)
Supplement: Supplementary file 1 [file pathogens-11-00689-s001.zip › pathogens-1705715-supplementary.pdf]

Table S1 - Characteristics of the *S. pseudintermedius* and CoNS isolated from pets, livestock and wild animals.

Isolate	Animal	Staphylococci species	Infection/ colonization	Resistance		Virulence factors
				Phenotype	Genotype	
VS2733	Wild hare	*S. cohnii spp urealyticus*	Colonization	PEN-FOX-ERY	*bla*Z, *erm*A, *erm*B	-
VS2734	Wild hare	*S. lentus*	Colonization	Susceptible	*Susceptible*	-
VS2735	Wild hare	*S. vitulinus*	Colonization	Susceptible	*Susceptible*	-
VS2736	Wild hare	*S. vitulinus*	Colonization	Susceptible	*Susceptible*	-
VS2737	Wild hare	*S. sciuri*	Colonization	PEN	*bla*Z	-
VS2738	Wild hare	*S. sciuri*	Colonization	PEN	-	-
VS2739	Wild hare	*S. sciuri*	Colonization	PEN	-	-
VS2740	Wild hare	*S. sciuri*	Colonization	PEN	-	-
VS2741	Wild hare	*S. sciuri*	Colonization	PEN	-	-
VS2742	Wild hare	*S. sciuri*	Colonization	PEN	-	-
VS2743	Wild hare	*S. sciuri*	Colonization	PEN	-	-
VS2744	Wild hare	*S. sciuri*	Colonization	PEN	-	-
VS2777	Dog	*S. pseudintermedius*	Infection (pyoderma)	PEN	*bla*Z	-
VS2778	Dog	*S. pseudintermedius*	Infection (pyoderma)	PEN-CIP-CN-TOB-KAN-STR-ERY-CD-TET-SXT	*bla*Z, *erm*B, *aph*(3´)-IIIa	-
VS2779	Dog	*S. pseudintermedius*	Infection (pyoderma)	PEN-KAN-STR-ERY-CD-TET-CHL	*bla*Z, *erm*B, *aph*(3´)-IIIa, *tet*M	-
VS2780	Dog	*S. pseudintermedius*	Infection (pyoderma)	PEN-CIP-CN-TOB-KAN-STR-ERY-CD-TET-SXT	*bla*Z, *erm*B, *aac*(6')-Ie-*aph*(2'')-Ia, *aph*(3´)-IIIa, *tet*M	-

VS2781	Dog	*S. pseudintermedius*	Infection (pyoderma)	PEN-CN-TOB-KAN-STR-ERY-CD-TET-SXT	*bla*Z, *erm*B, *aac*(6′)-Ie-*aph*(2′′)-Ia, *tet*M	-
VS2782	Dog	*S. pseudintermedius*	Infection (pyoderma)	PEN-KAN-STR-ERY-CD-TET-SXT	*bla*Z, *erm*B, *aph*(3′)-IIIa, *tet*M	-
VS2783	Dog	*S. pseudintermedius*	Infection (pyoderma)	PEN-CIP-CN-TOB-KAN-STR-ERY-CD-TET-CHL-SXT	*bla*Z, *msr*(A/B), *aph*(3′)-IIIa, *aac*(6′)-Ie-*aph*(2′′)-Ia, *tet*M	-
VS2784	Dog	*S. pseudintermedius*	Infection (pyoderma)	PEN-CIP-CN-TOB-KAN-STR-ERY-CD-SXT	*bla*Z, *erm*B, *aac*(6′)-Ie-*aph*(2′′)-Ia, *aph*(3′)-IIIa	-
VS2785	Dog	*S. pseudintermedius*	Infection (pyoderma)	PEN-CIP-KAN-STR-ERY-CD-TET-CHL-SXT	*bla*Z, *erm*B, *tet*M	-
VS2786	Dog	*S. pseudintermedius*	Infection (pyoderma)	PEN-CIP-CN-TOB-KAN-STR-ERY-CD-TET-SXT	*bla*Z, *erm*B, *aph*(3′)-IIIa, *tet*M	-
VS2787	Dog	*S. pseudintermedius*	Infection (pyoderma)	PEN-CIP-KAN-STR-ERY-CD-SXT-RD	*bla*Z, *erm*B, *aph*(3′)-IIIa	-
VS2788	Dog	*S. pseudintermedius*	Infection (pyoderma)	PEN-CIP-KAN-STR-ERY-CD-SXT-RD	*bla*Z, *erm*B, *aph*(3′)-IIIa	-
VS2789	Dog	*S. pseudintermedius*	Infection (pyoderma)	PEN-CIP-CN-TOB-KAN-STR-ERY-CD-TET-SXT	*bla*Z, *erm*B, *msr*(A/B), *aph*(3′)-IIIa, *tet*M	-
VS2790	Dog	*S. pseudintermedius*	Infection (pyoderma)	PEN-CIP-CN-TOB-KAN-STR-ERY-CD-TET-CHL-SXT	*bla*Z, *erm*B, *aac*(6′)-Ie-*aph*(2′′)-Ia, *aph*(3′)-IIIa, *tet*M	-
VS2791	Dog	*S. pseudintermedius*	Infection (pyoderma)	PEN-CIP-CN-TOB-KAN-STR-ERY-CD-TET-CHL-SXT	*bla*Z, *erm*B, *msr*(A/B), *aac*(6′)-Ie-*aph*(2′′)-Ia, *aph*(3′)-IIIa, *tet*M	-
VS2792	Dog	*S. pseudintermedius*	Infection (pyoderma)	PEN-CIP-CN-TOB-KAN-STR-ERY-CD-TET-SXT	*bla*Z, *erm*B, *aac*(6′)-Ie-*aph*(2′′)-Ia, *aph*(3′)-IIIa, *tet*M	-
VS2793	Dog	*S. pseudintermedius*	Infection (pyoderma)	PEN-CIP-CN-TOB-KAN-STR-ERY-CD-TET-SXT	*bla*Z, *erm*B, *aac*(6′)-Ie-*aph*(2′′)-Ia, *aph*(3′)-IIIa, *tet*M	-
VS2794	Dog	*S. pseudintermedius*	Infection (pyoderma)	PEN-CIP-CN-TOB-KAN-STR-ERY-CD-TET-SXT	*bla*Z, *erm*B, *aac*(6′)-Ie-*aph*(2′′)-Ia, *aph*(3′)-IIIa	-

VS2795	Dog	*S. pseudintermedius*	Infection (pyoderma)	PEN-CIP-CN-TOB-KAN-STR-ERY-CD-TET-SXT	*bla*Z, *erm*B, *aac*(6′)-Ie-*aph*(2′′)-Ia, *aph*(3′)-IIIa, *tet*M	-
VS2796	Dog	*S. pseudintermedius*	Infection (pyoderma)	PEN-CIP-CN-TOB-KAN-STR-ERY-CD-TET-CHL-SXT	*bla*Z, *erm*B, *aac*(6′)-Ie-*aph*(2′′)-Ia, *aph*(3′)-IIIa, *tet*M	-
VS2797	Dog	*S. pseudintermedius*	Infection (pyoderma)	PEN-CN-TOB-KAN-STR-ERY-CD-TET-SXT-RD	*bla*Z, *erm*B, *aph*(3′)-IIIa	-
VS2798	Dog	*S. pseudintermedius*	Infection (pyoderma)	PEN-CIP-CN-TOB-KAN-STR-ERY-CD-FD-SXT	*bla*Z, *erm*B, *aac*(6′)-Ie-*aph*(2′′)-Ia, *aph*(3′)-IIIa	-
VS2799	Dog	*S. pseudintermedius*	Infection (pyoderma)	PEN-CN-KAN-ERY-CD-TET-SXT	*bla*Z, *erm*B, *msr*(A/B), *aac*(6′)-Ie-*aph*(2′′)-Ia, *aph*(3′)-IIIa	-
VS2800	Dog	*S. pseudintermedius*	Infection (pyoderma)	PEN-CIP-CN-TOB-KAN-STR-ERY-CD-TET-SXT	*bla*Z, *erm*B, *msr*(A/B), *aac*(6′)-Ie-*aph*(2′′)-Ia, *aph*(3′)-IIIa	-
VS2801	Dog	*S. pseudintermedius*	Infection (pyoderma)	PEN-CIP-CN-TOB-KAN-STR-ERY-CD-TET-SXT	*bla*Z, *erm*B, *msr*(A/B), *aac*(6′)-Ie-*aph*(2′′)-Ia, *aph*(3′)-IIIa	-
VS2802	Dog	*S. pseudintermedius*	Infection (pyoderma)	PEN-CIP-CN-TOB-KAN-STR-ERY-CD-TET-SXT	*bla*Z, *erm*B, *aac*(6′)-Ie-*aph*(2′′)-Ia, *aph*(3′)-IIIa, *tet*M	-
VS2803	Dog	*S. pseudintermedius*	Infection (pyoderma)	PEN-CIP-CN-TOB-KAN-STR-ERY-CD-TET-SXT	*bla*Z, *erm*B, *aac*(6′)-Ie-*aph*(2′′)-Ia, *aph*(3′)-IIIa	-
VS2804	Dog	*S. pseudintermedius*	Infection (pyoderma)	PEN-CN-TOB-KAN-STR-ERY-CD-TET-SXT	*bla*Z, *erm*B, *msr*(A/B), *aph*(3′)-IIIa	-
VS2805	Dog	*S. pseudintermedius*	Infection (pyoderma)	PEN-CIP-CN-TOB-KAN-STR-ERY-CD-TET-SXT	*erm*B, *msr*(A/B), *aac*(6′)-Ie-*aph*(2′′)-Ia, *aph*(3′)-IIIa	-
VS2806	Dog	*S. pseudintermedius*	Infection (pyoderma)	PEN-CIP-CN-TOB-KAN-STR-ERY-CD-TET-SXT	*bla*Z, *erm*B, *aac*(6′)-Ie-*aph*(2′′)-Ia, *aph*(3′)-IIIa, *tet*M	-
VS2807	Dog	*S. pseudintermedius*	Infection (pyoderma)	PEN-CIP-CN-TOB-KAN-STR-ERY-CD-TET-SXT	*bla*Z, *erm*B, *aac*(6′)-Ie-*aph*(2′′)-Ia, *aph*(3′)-IIIa, *tet*M	-
VS3185	Dog	*S. pseudintermedius*	Colonization	PEN, FOX	*mec*A	*luk*S/F-I, *siet*
VS3186	Dog	*S. cohnii*	Colonization	PEN	*mec*A	-
VS3192	Dog	*S. lentus*	Colonization	PEN	*mec*A	-

VS3196	Dog	*S. pseudintermedius*	Colonization	PEN, FOX	*mec*A, *bla*Z	*luk*S/F-I
VS3198	Dog	*S. pseudintermedius*	Colonization	PEN, FOX, CN, TOB, KAN	*mec*A, *blaZ*, *aac*(6′)-Ie-*aph*(2′′)-Ia, *aph*(3′)-IIIa , *str*	*luk*S/F-I, *siet*
VS3200	Dog	*S. pseudintermedius*	Colonization	PEN, FOX	*mec*A, *bla*Z	*luk*S/F-I, *siet*
VS3204	Dog	*S. sciuri*	Colonization	PEN	*bla*Z, *mec*A	
VS3206	Dog	*S. pseudintermedius*	Colonization	PEN, FOX	*mec*A, *bla*Z	lukS/F-I, *siet*
VS3207	Dog	*S. lentus*	Colonization	PEN	*mec*A	-
VS3209	Dog	*S. vitulinus*	Colonization	PEN	*mec*A	-
VS3211	Dog	*S. lentus*	Colonization	PEN, CN, KAN, CD, C	*mec*A, *aac*(6′)-Ie-*aph*(2′′)-Ia, *aph*(3′)-IIIa, *mph*C, cat_{p221}	*hla*
VS3213	Dog	*S. sciuri*	Colonization	PEN	*mec*A	-
VS3215	Dog	*S. lentus*	Colonization	PEN	*mec*A, *bla*Z	-
VS3216	Dog	*S. lentus*	Colonization	PEN	*mec*A, *bla*Z,	-
VS2985	Owl	*S. epidermidis*	Colonization	PEN, FD	*bla*Z, *fus*B	-
VS2986	Owl	*S. epidermidis*	Colonization	PEN, ERY, FD	*bla*Z, *msr*(A/B), *mph*C, *fus*B	-
VS2987	Owl	*S. sciuri*	Colonization	Susceptible	-	-
VS2988	Owl	*S. sciuri*	Colonization	Susceptible	-	-
VS2989	Owl	*S. sciuri*	Colonization	PEN	*mec*A	-
VS2990	Owl	*S. sciuri*	Colonization	PEN	*mec*A	-
VS2991	Owl	*S. sciuri*	Colonization	PEN, CD, TET, FD	*mec*A, *mph*C, *tet*K	-
VS2992	Owl	*S. sciuri*	Colonization	PEN, CD, FD	-	-
VS2993	Owl	*S. sciuri*	Colonization	PEN, FOX, CD	*mec*A, *mph*C	-

VS2994	Owl	*S. sciuri*	Colonization	PEN	*mec*A	-
VS2995	Owl	*S. sciuri*	Colonization	PEN, CD, FD	-	-
VS2996	Owl	*S. sciuri*	Colonization	Susceptible	-	-
VS2997	Owl	*S. sciuri*	Colonization	Susceptible	-	-
VS2998	Owl	*S. sciuri*	Colonization	Susceptible	-	-
VS2999	Owl	*S. sciuri*	Colonization	PEN, FD	-	-
VS3000	Owl	*S. sciuri*	Colonization	PEN, FOX, CD, FD	*mec*A, *mph*C	-
VS3001	Owl	*S. sciuri*	Colonization	Susceptible	-	-
VS3002	Owl	*S. sciuri*	Colonization	PEN	-	-
VS3003	Owl	*S. sciuri*	Colonization	PEN, FD	*mec*A	-
VS3004	Owl	*S. sciuri*	Colonization	PEN, FOX, CD, TET, FD	*mec*A	-
VS3005	Owl	*S. sciuri*	Colonization	Susceptible	-	-
VS3006	Owl	*S. sciuri*	Colonization	Susceptible	-	-
VS3007	Owl	*S. lentus*	Colonization	CD	*mph*C	-
VS3008	Owl	*S. lentus*	Colonization	PEN, CD, TET, FD	*tet*K	-
VS3009	Owl	*S. lentus*	Colonization	PEN, CD, TET	*tet*K	-
VS3010	Owl	*S. lentus*	Colonization	CD, TET	*mph*C	-
VS3011	Owl	*S. lentus*	Colonization	Susceptible	-	-
VS3012	Owl	*S. lentus*	Colonization	Susceptible	-	-
VS3013	Owl	*S. lentus*	Colonization	TET	*tet*K	-
VS3014	Owl	*S. lentus*	Colonization	Susceptible	-	-

VS3015	Owl	*S. lentus*	Colonization	PEN, CD, FD	*mec*A	-
VS3016	Owl	*S. lentus*	Colonization	FD	-	-
VS3017	Owl	*S. lentus*	Colonization	Susceptible	*mec*A	-
VS3018	Owl	*S. vitulinus*	Colonization	Susceptible	*mec*A	-
VS3019	Owl	*S. vitulinus*	Colonization	PEN, FD	*mec*A	-
VS3020	Owl	*S. haemolyticus*	Colonization	Susceptible	-	-
VS3021	Owl	*S. haemolyticus*	Colonization	PEN, FOX, CIP, ERY, CD, TET, SXT	*mph*C, *msr*(A/B), *tet*O	-
VS3022	Owl	*S. saprophyticus*	Colonization	Susceptible	*mec*A	-
VS3023	Owl	*S. xylosus*	Colonization	PEN, FOX, ERY, CD, TET, C, FD	*mph*C, *tet*M, *tet*L	-
VS3024	Owl	*S. xylosus*	Colonization	ERY	-	-
VS3025	Owl	*S. succinus*	Colonization	PEN	-	-
VS3026	Homebred chicken	*S. lentus*	Colonization	CIP, TOB, KAN, ERY, CD, TET	*erm*B, *aph*(3′)-IIIa, *ant*(4′)-Ia, *tet*L	-
VS3027	Homebred chicken	*S. lentus*	Colonization	CIP, TOB, ERY, CD, TET	*erm*C, *ant*(4′)-Ia, *tet*K	-
VS3028	Homebred chicken	*S. lentus*	Colonization	CIP, TOB, KAN, ERY, CD, SXT	*erm*B, *aph*(3′)-IIIa, *ant*(4′)-Ia, *dfr*K	-
VS3029	Homebred chicken	*S. lentus*	Colonization	CIP, TOB, KAN, ERY, CD	*erm*C, *mph*C, *aph*(3′)-IIIa, *ant*(4′)-Ia	-
VS3030	Homebred chicken	*S. lentus*	Colonization	CIP, ERY, CD, C, SXT	*erm*A, *erm*C, *mph*C, *dfr*D, *dfr*K, cat_{p194}	-
VS3031	Homebred chicken	*S. lentus*	Colonization	CIP, ERY, CD	*mph*C	-
VS3032	Homebred chicken	*S. lentus*	Colonization	PEN, CIP, ERY, CD, TET	*erm*C, *tet*L	-

VS3033	Homebred chicken	*S. lentus*	Colonization	PEN, CIP, ERY, CD, TET	*erm*C, *mph*C, *tet*K	-
VS3034	Homebred chicken	*S. lentus*	Colonization	PEN, TOB, KAN, ERY, CD, TET, FD	*erm*B, *erm*C, *mph*C, *aph*(3′)-IIIa, *ant*(4′)-Ia, *tet*L, *tet*K	-
VS3035	Homebred chicken	*S. lentus*	Colonization	PEN, CIP, ERY, CD	*erm*C, *mph*C	-
VS3036	Homebred chicken	*S. sciuri*	Colonization	PEN, TOB, KAN, ERY, CD, TET	*erm*B, *aph*(3′)-IIIa, *ant*(4′)-Ia, *tet*L, *tet*K	-
VS3037	Commercial chicken	*S. lentus*	Colonization	CIP, ERY, CD, TET	*mph*C, *tet*L	-
VS3038	Commercial chicken	*S. lentus*	Colonization	CIP, ERY, CD, FD	*erm*A, *erm*C, *mph*C	-
VS3039	Commercial chicken	*S. lentus*	Colonization	PEN, CIP, ERY, CD	*erm*C	-
VS3040	Commercial chicken	*S. lentus*	Colonization	PEN, FOX, CIP, CN, TOB, KAN, ERY, CD, TET	*erm*A, *erm*C, *aph*(3′)-IIIa, *tet*L, *tet*M	-
VS3041	Commercial chicken	*S. lentus*	Colonization	PEN, FOX+D19:D21, ERY, CD, FD	*erm*C, *mph*C	-
VS3042	Commercial chicken	*S. lentus*	Colonization	PEN, CIP, ERY, CD	*erm*A, *erm*C, *mph*C,	-
VS3043	Commercial chicken	*S. lentus*	Colonization	PEN, CIP, ERY, CD	*erm*A, *erm*B, *erm*C, *mph*C	-
VS3044	Commercial chicken	*S. lentus*	Colonization	PEN, ERY, CD	*erm*C, *mph*C	-
VS3045	Commercial chicken	*S. lentus*	Colonization	PEN, ERY, CD, TET	*erm*A, *erm*C, *mph*C, *tet*L, *tet*M,	-
VS3046	Commercial chicken	*S. lentus*	Colonization	PEN, FOX, CIP, TOB, KAN, TET, C, SXT	*erm*A, *erm*C, *mph*C, *aph*(3′)-IIIa, *ant*(4′)-Ia, *tet*L, *dfr*D, *dfr*K, *fex*A	-
VS3047	Commercial chicken	*S. lentus*	Colonization	PEN, ERY, CD, TET	*erm*C, *mph*C, *tet*L	-

VS3048	Commercial chicken	*S. cohnii* spp. *urealyticus*	Colonization	PEN, FOX, ERY, CD, TET, FD	*erm*B, *mph*C	-
VS3049	Commercial chicken	*S. cohnii* spp. *urealyticus*	Colonization	PEN, ERY, CD, TET	*erm*A, *erm*C, *mph*C, *tet*L, *tet*M, *tet*K	-
VS3050	Quail	*S. lentus*	Colonization	PEN, TOB, ERY, CD, TET, FD	*erm*A, *erm*B, *mph*C, *aph*(3′)-IIIa, *ant*(4′)-Ia, *tet*L, *tet*K	-
VS3051	Quail	*S. lentus*	Colonization	PEN, FOX, CIP, CN, TOB, KAN, ERY, CD, TET, FD	*erm*C, *mph*C, *str*, *tet*K, *tet*O	-
VS3052	Quail	*S. lentus*	Colonization	PEN, CIP, ERY, CD, TET, FD, SXT	*erm*C, *mph*C, *tet*L, *dfr*K	-
VS3053	Quail	*S. lentus*	Colonization	CIP, ERY, CD, TET, C, FD	*mec*A, *erm*C, *mph*C,	-
VS3054	Quail	*S. lentus*	Colonization	PEN, CIP, ERY, CD, TET, FD	*erm*C, *mph*C, *tet*K	-
VS3055	Quail	*S. lentus*	Colonization	PEN, CIP, ERY, CD, TET, FD	*erm*C, *mph*C, *tet*K, *tet*L	-
VS3056	Quail	*S. lentus*	Colonization	PEN, CIP, ERY, CD, TET, FD	*erm*C, *mph*C, *tet*K	-
VS3057	Quail	*S. lentus*	Colonization	PEN, CIP, ERY, CD, TET, C, FD, SXT	*erm*C, *mph*C, *tet*L, *dfr*K	-
VS3058	Quail	*S. lentus*	Colonization	PEN, CIP, TOB, ERY, CD, TET	*erm*B, *mph*C, *ant*(4′)-Ia, *tet*L, *tet*K	-
VS3059	Quail	*S. lentus*	Colonization	PEN, ERY, CD, TET, FD	*erm*C, *tet*L, *tet*K	-
VS3060	Quail	*S. lentus*	Colonization	PEN, CIP, TOB, ERY, CD, TET	*erm*B, *mph*C, *ant*(4′)-Ia, *tet*L, *tet*K	-
VS3061	Quail	*S. lentus*	Colonization	PEN, TOB, ERY, CD, TET	*erm*C, *mph*C, *ant*(4′)-Ia, *tet*L, *tet*K	-
VS3062	Quail	*S. lentus*	Colonization	PEN, TOB, KAN, ERY, CD, TET	*erm*C, *mph*C, *aph*(3′)-IIIa, *ant*(4′)-Ia, *tet*K, *tet*L	-
VS3063	Quail	*S. lentus*	Colonization	PEN, ERY, CD, TET	*erm*C, *mph*C, *tet*L, *tet*K	-

VS3064	Quail	*S. lentus*	Colonization	PEN, CIP, TOB, KAN, ERY, CD, TET, SXT	*erm*B, *mph*C, *aph*(3′)-IIIa, *ant*(4′)-Ia, *str*, *tet*L, *dfr*K	-
VS3065	Quail	*S. sciuri*	Colonization	PEN, TOB, ERY, CD, TET, FD	*erm*B, *ant*(4′)-Ia, *tet*L, *tet*K	-
VS3066	Quail	*S. sciuri*	Colonization	PEN, TOB, KAN, ERY, CD, TET, FD	*erm*B, *aph*(3′)-IIIa, *ant*(4′)-Ia, *tet*L, *tet*K, *tet*O	-
VS3067	Quail	*S. sciuri*	Colonization	PEN, FOX, CD, TET, FD	*tet*K	-
VS3068	Quail	*S. sciuri*	Colonization	PEN, CIP, TOB, ERY, CD, TET, FD	*erm*C, *tet*L, *tet*K	-
VS3069	Quail	*S. sciuri*	Colonization	PEN, CIP, TOB, ERY, CD, TET, FD	*erm*C, *ant*(4′)-Ia, *str*	-
VS3070	Quail	*S. sciuri*	Colonization	PEN, TOB, KAN, ERY, CD, TET, FD	*erm*B, *mph*C, *ant*(4′)-Ia, *ant*(4′)-Ia, *tet*L, *tet*K	-
VS3071	Quail	*S. sciuri*	Colonization	PEN, CIP, ERY, CD, TET	*erm*C, *mph*C, *tet*K	-
VS3072	Quail	*S. sciuri*	Colonization	PEN, FOX, ERY, CD, TET, C, FD	*erm*B, *erm*C, *mph*C, *tet*L, *tet*K, *tet*M	-
VS3073	Quail	*S. sciuri*	Colonization	PEN, TOB, ERY, CD, TET	*erm*B, *mph*C, *ant*(4′)-Ia, *tet*L, *tet*K	-
VS3074	Quail	*S. sciuri*	Colonization	PEN, CIP, TOB, KAN, ERY, CD, TET, SXT	*erm*C, *mph*C, *aph*(3′)-IIIa, *ant*(4′)-Ia, *tet*K	-
VS3075	Quail	*S. sciuri*	Colonization	ERY, CD, TET, SXT	*ermB*, *mph*C, *tet*L, *dfr*K	-
VS3076	Quail	*S. sciuri*	Colonization	PEN, FOX, ERY, CD, TET, FD	*erm*B, *erm*C, *mph*C, *tet*L, *tet*K, *tet*O, *tet*M	-
VS3077	Quail	*S. sciuri*	Colonization	PEN, FOX, LNZ, ERY, CD, TET, C, FD	*cfr*, *erm*B, *erm*C, *mph*C, *tet*L, *tet*K, *tet*M	-
VS3078	Quail	*S. sciuri*	Colonization	PEN, FOX, CD, TET, FD	*mph*C, *tet*L	-
VS3079	Quail	*S. cohnii* spp. *urealyticus*	Colonization	PEN, FOX, ERY, CD, TET	*erm*C, *mph*C, *tet*L, *tet*K,	-

VS3080	Quail	*S. cohnii* spp. *urealyticus*	Colonization	PEN, FOX, CIP, CN, ERY, CD, TET, C, FD	*erm*B, *erm*C, *mph*C, *tet*K, *tet*O	-
VS3081	Quail	*S. cohnii* spp. *urealyticus*	Colonization	PEN, FOX, CN, TOB, ERY, CD, TET, FD	*erm*B, *erm*C, *mph*C, *ant*(4′)-Ia, *str*, *tet*K, *tet*L, *tet*O	-
VS3082	Quail	*S. cohnii* spp. *urealyticus*	Colonization	PEN, FOX, ERY, CD, TET, FD	*erm*C, *tet*L, *tet*K, *tet*O	-
VS3083	Quail	*S. cohnii* spp. *urealyticus*	Colonization	PEN, TOB, KAN, ERY, CD, TET	*erm*C, *mph*C, *aph*(3′)-IIIa, *ant*(4′)-Ia, *tet*K, *tet*L	-
VS3084	Quail	*S. cohnii* spp. *urealyticus*	Colonization	PEN, FOX, ERY, CD, TET, FD	*erm*B, *mph*C, *tet*L, *tet*K, *tet*O, *tet*M	-
VS3085	Quail	*S. cohnii* spp. *urealyticus*	Colonization	PEN, FOX, ERY, CD, TET, FD	*erm*C, *mph*C, *tet*L, *tet*K	-
VS3086	Quail	*S. cohnii* spp. *urealyticus*	Colonization	PEN, FOX, CIP, CN, TOB, KAN, ERY, CD, TET, FD	*erm*B, *erm*C, *mph*C, *aph*(3′)-IIIa	-
VS3087	Quail	*S. cohnii* spp. *urealyticus*	Colonization	PEN, FOX, ERY, CD, TET, FD	*erm*B, *erm*C, *mph*C, *tet*L, *tet*K, *tet*O, *tet*M	-
VS3088	Quail	*S. cohnii* spp. *urealyticus*	Colonization	PEN, FOX, ERY, CD, TET, FD	*erm*C, *mph*C, *tet*L, *tet*K, *tet*O	-
VS3089	Quail	*S. cohnii* spp. *urealyticus*	Colonization	PEN, FOX, ERY, CD, TET, FD	*erm*B, *erm*C, *mph*C, *tet*L, *tet*K, *tet*O	-
VS3090	Quail	*S. cohnii* spp. *urealyticus*	Colonization	PEN, FOX, CN, TOB, KAN, ERY, CD, TET, FD	*erm*C, *mph*C, *aph*(3′)-IIIa, *str*, *tet*L, *tet*K, *tet*O	-
VS3091	Quail	*S. cohnii* spp. *urealyticus*	Colonization	PEN, FOX, ERY, CD, TET, FD	*erm*C, *mph*C, *tet*L, *tet*K, *tet*O	-
VS3092	Quail	*S. cohnii* spp. *urealyticus*	Colonization	PEN, FOX, ERY, CD, TET	*erm*C, *mph*C	-
VS3093	Quail	*S. cohnii* spp. *urealyticus*	Colonization	PEN, FOX, ERY, CD, TET, FD	*erm*C, *tet*L, *tet*K, *tet*O	-
VS3094	Quail	*S. cohnii* spp. *urealyticus*	Colonization	PEN, FOX, TOB, KAN, ERY, CD, TET, C, FD	*erm*C, *aph*(3′)-IIIa, *tet*L, *tet*K	-

VS3095	Quail	*S. cohnii* spp. *urealyticus*	Colonization	PEN, FOX, ERY, CD, TET, FD	*erm*C, *mph*C, *tet*L, *tet*K, *tet*O, *tet*M	-
VS3096	Quail	*S. cohnii* spp. *urealyticus*	Colonization	PEN, FOX, TOB, KAN, ERY, CD, TET, C, FD	*erm*C, *aph*(3′)-IIIa, *tet*L, *tet*K, *tet*O	-
VS3097	Quail	*S. cohnii* spp. *urealyticus*	Colonization	PEN, CIP, ERY, CD, TET, FD	*erm*C, *tet*L, *tet*K, *tet*O	-
VS3098	Quail	*S. haemolyticus*	Colonization	PEN, CIP, TOB, TET, FD	*aph*(3′)-IIIa, *tet*L	-
VS3099	Quail	*S. haemolyticus*	Colonization	PEN, CIP, TOB, KAN, ERY, CD, TET, SXT	*erm*C, *mph*C, *aph*(3′)-IIIa, *ant*(4′)-Ia, *str*, *tet*L, *dfr*K	-
VS3100	Quail	*S. haemolyticus*	Colonization	PEN, FOX, ERY, CD, TET, FD	*erm*B, *erm*C, *mph*C, *tet*L, *tet*K	-
VS3119	Donkey	*S. lentus*	Colonization	PEN, CIP, ERY, CD, TET, FD	*mec*A, *bla*Z, *lnu*A	-
VS3120	Donkey	*S. xylosus*	Colonization	TET		-
VS3121	Donkey	*S. vitulinus*	Colonization	PEN, TET	*mec*A	-
VS3122	Donkey	*S. sciuri*	Colonization	CN, TOB, KAN, FD	*mec*A, *aph*(3′)-IIIa, *ant*(4′)-Ia, *str*	-
VS3123	Donkey	*S. sciuri*	Colonization	PEN, CD, FD	*mec*A	-
VS3124	Donkey	*S. sciuri*	Colonization	FD	*mec*A	-
VS3125	Donkey	*S. sciuri*	Colonization	PEN, FOX, LNZ, CN, TOB, KAN, ERY, CD, TET, FD	*mec*A, *aph*(3′)-IIIa	-
VS3126	Donkey	*S. sciuri*	Colonization	PEN, FOX, LNZ, ERY, LNZ, CN, TOB, KAN, CD	*mec*A, *optr*A, *erm*B, *aph*(3′)-IIIa	*eta*
VS3127	Donkey	*S. sciuri*	Colonization	PEN, FOX, CD, FD	*mec*A	-
VS3128	Donkey	*S. sciuri*	Colonization	PEN, CN, CD, FD	*mec*A, *aph*(3′)-IIIa	-
VS3130	Donkey	*S. sciuri*	Colonization	Susceptible		-
VS3131	Donkey	*S. sciuri*	Colonization	Susceptible		-

VS3132	Donkey	*S. sciuri*	Colonization	Susceptible	*mec*A	-
VS3133	Donkey	*S. sciuri*	Colonization	Susceptible	*mec*A	-
VS3134	Donkey	*S. sciuri*	Colonization	Susceptible	*mec*A	-
VS3135	Donkey	*S. sciuri*	Colonization	Susceptible	*mec*A	*hla*
VS3136	Donkey	*S. sciuri*	Colonization	TET	*mec*A, *tet*K	-
VS3137	Donkey	*S. sciuri*	Colonization	Susceptible	*mec*A	-
VS3138	Donkey	*S. sciuri*	Colonization	TET	*mec*A, *aph*(3')-IIIa, *ant*(4')-Ia, *str*, *tet*M	-
VS3139	Donkey	*S. sciuri*	Colonization	CD, FD	*mec*A	-
VS3151	Camel	*S. chromogenes*	Colonization	PEN	*mec*A	-
VS3152	Camel	*S. epidermidis*	Colonization	PEN	*mec*A	*hla, hld*
VS3153	Camel	*S. hominis*	Colonization	PEN	*mec*A	-
VS3154	Camel	*S. lentus*	Colonization	PEN, ERY, CD	*mec*A, *mph*C	*hla*
VS3155	Camel	*S. lentus*	Colonization	PEN	*mec*A	-
VS3156	Camel	*S. lentus*	Colonization	PEN, ERY, CD, FD	*mec*A	-
VS3157	Camel	*S. lentus*	Colonization	PEN	*mec*A	-
VS3158	Camel	*S. lentus*	Colonization	PEN	*mec*A	-
VS3159	Camel	*S. lentus*	Colonization	PEN	*mec*A	-
VS3160	Camel	*S. lentus*	Colonization	PEN	*mec*A	-
VS3161	Camel	*S. lentus*	Colonization	PEN	*mec*A	-
VS3162	Camel	*S. lentus*	Colonization	PEN	*mec*A	-

VS3163	Camel	*S. lentus*	Colonization	PEN, FD	*mec*A	*hld*
VS3164	Camel	*S. lentus*	Colonization	PEN, FD	*mec*A	-
VS3165	Camel	*S. lentus*	Colonization	PEN	*mec*A	-
VS3166	Camel	*S. lentus*	Colonization	PEN	*mec*A, *mph*C	-
VS3167	Camel	*S. sciuri*	Colonization	PEN	*mec*A	-
VS3168	Camel	*S. sciuri*	Colonization	PEN	*mec*A	-
VS3169	Camel	*S. sciuri*	Colonization	PEN	*mec*A	-
VS3170	Camel	*S. sciuri*	Colonization	PEN	*mec*A	-
VS3171	Camel	*S. sciuri*	Colonization	PEN	*mec*A	-
VS3172	Camel	*S. sciuri*	Colonization	PEN	*mec*A	-
VS3173	Camel	*S. sciuri*	Colonization	PEN	*mec*A	*hld*
VS3174	Camel	*S. sciuri*	Colonization	PEN	*mec*A	-
VS3175	Camel	*S. sciuri*	Colonization	PEN	*mec*A	-
VS3176	Camel	*S. sciuri*	Colonization	PEN	*mec*A	*hld*
VS3177	Camel	*S. sciuri*	Colonization	PEN	*mec*A	*hld*
VS3178	Camel	*S. sciuri*	Colonization	PEN, CD, FD	*mec*A	-
VS3179	Camel	*S. xylosus*	Colonization	PEN	*mec*A	-
VS3180	Camel	*S. xylosus*	Colonization	PEN	*mec*A	-
VS3181	Camel	*S. xylosus*	Colonization	PEN	*mec*A	*hld*

Abbreviations. PEN: penicillin; CIP: ciprofloxacin; FOX: cefoxitin; CN: gentamycin; TOB: tobramycin; KAN: kanamycin; STR: streptomycin; ERY: erythromycin; CD: clindamycin; TET: tetracycline; SXT: sulfamethoxazole-trimethoprim; C: chloramphenicol.
